# Evaluating the role of clinical history and laboratorial tests in IgE mediated cow's milk allergy diagnosis

**DOI:** 10.1186/1939-4551-8-S1-A124

**Published:** 2015-04-08

**Authors:** Beni Morgenstern, Cleonir Beck, Cristina Jacob, Andrea Gushken, Antonio Carlos Pastorino, Ulisses Doria, Mayra Dorna, Glauce Yonamine, Ana Paula Moschione Castro

**Affiliations:** 1 Unit of Allergy and Immunology, Brazil; 2 Instituto Da Criança, Brazil; 3 Nutritionist of Division of Nutrition, Brazil

## Background

IgE mediated cow´s milk allergy (CMA) is a frequent disease in pediatric population, and challenge tests are considered gold standard in its diagnosis, although its use is limited in clinical practice. An appropriated anamnesis and laboratorial analysis are the most frequent and feasible used tools. The aim of this study was to evaluate the role of these practices in IgE mediated CMA diagnosis.

## Methods

It was a retrospective study based on patient data charts from a reference center in food allergy. All patients who performed open, single or double blind placebo controlled oral food challenges (OFC) for CMA diagnosis were included. Clinical history was defined as suggestive when patients presented at any time anaphylaxis, urticaria, angioedema, laryngeal edema and dyspnea. Diarrhea, vomiting, cough, wheezing, rhinoconjunctivitis, pruritus and erythema were considered as dubious. Symptoms onset <2 hours after food ingestion was also considered suggestive. Dubious and/or subjective symptoms or delayed ones were considered undetermined. Specific IgE (sIgE) was included when evaluated ± 12 months from OFC. Skin prick test was considered positive when wheal was >3mm and/or serum specific IgE >0.35 kU/L. Sensitivity (Se), specificity (Sp), positive and negative predictive value (PPV and NPV) and likelihood ratio (LR) were established, comparing anamnesis, anaphylaxis, laboratorial tests *versus* OFC.

## Results

92 patients were included (43 OFC+; 49 OFC-). The median age at challenge test was 2.5 years (0.4-10.7) (OFC+ 3.0y; OFC- 2.4y). The median time between symptoms onset and food challenge was 2.3 years (0.2-10.4) (OFC+ 2.7; OFC- 2.2). Suggestive clinical history was present in 93% of patients in OFC+, compared to 61% of patients in OFC- (Se 93%; Sp 38%; PPV 57%; NPV 86%; LR 1.51; p<0.05). Considering only anaphylactic symptoms versus OFC the results were Se 44%; Sp 87%; PPV 76%; NPV 64% and LR 3.06 (p<0.05). Specific IgE was present in 100% of patients in OFC+ resulting in Se 100%; Sp 48%; PPV 63%; NPV 100% and LR 1.96 (p<0.05). Suggestive clinical history associated with symptoms onset <2 hours and presence of sIgE demonstrated a Se 88%; Sp 73%; PPV 74%; NPV 88% and LR 3.3 (p<0.05).

## Conclusions

Suggestive clinical history associated to positive sIgE was helpful on the diagnosis of CMA. Generally, the OFC remains necessary to set or exclude diagnosis. Negative sIgE was useful to exclude CMA.

